# No exotic matter in asteroids

**DOI:** 10.1140/epjp/s13360-026-07415-z

**Published:** 2026-02-17

**Authors:** Alan E. Rubin, Thomas H. Burbine

**Affiliations:** 1 Department of Earth, Planetary, and Space Sciences, University of California, Los Angeles, CA 90095-1567 USA; 2 Maine Mineral and Gem Museum, 99 Main Street, P.O. Box 500, Bethel, ME 04217 USA; 3 Department of Physics and Astronomy, Mount Holyoke College, South Hadley, MA 01075 USA

## Abstract

Solar System bodies have similar abundances of non-volatile elements. Asteroids are categorized as unmelted chondritic bodies or as differentiated bodies formed by extensive to global melting of chondritic progenitors. Reflectance spectra show asteroids are compositionally similar to meteorites (which are composed of non-exotic materials—mainly silicates, metallic Fe–Ni, sulfides, oxides, and organic matter). Dense refractory siderophile elements (e.g., Re, Os, Ir, Pt) are present in iron meteorites in total concentrations < 0.05 wt%. The upper limit on the density of an asteroid is ~ 8 g cm^−3^ for a zero-porosity core fragment composed of ~ 90 wt% Fe and ~ 10 wt% Ni. Carry (Planet Space Sci 73:98–118, 2012) compiled asteroid densities and included some anomalous values (caused by uncertainties in measurement) that he characterized as unrealistic and non-physical. One such value for asteroid (33) Polyhymnia (75.3 ± 9.7 g cm^−3^) was accepted by LaForge et al. (Eur Phys J Plus 138: 812, 2023) who characterized Polyhymnia as a compact ultradense object (CUDO) perhaps composed of stable superheavy elements (SHEs) or alpha matter (alpha particles in a Bose–Einstein condensate). Kiren et al. (Eur Phys J Plus 139: 547, 2024) proposed Polyhymnia could consist of degenerate dark matter. It is exceedingly unlikely that Polyhymnia or other asteroids contain exotic matter: (1) The listed bulk density of Polyhymnia is characterized as “unrealistic.” (2) Meteorites (~ 98.5% are from asteroids) are composed of non-exotic materials. (3) The spectra of “CUDO” asteroids do not differ from other asteroids of their taxonomic class. (4) SHEs and degenerate dark matter have not yet been shown to exist. (5) Alpha matter may occur naturally only in extreme astrophysical environments.

## Introduction

In his extensive compilation of asteroid bulk densities, Carry [1] reported some anomalous values caused by uncertainties in measurement; he characterized these values as “irrelevant” and “non-physical”. One such value was the bulk density of asteroid (33) Polyhymnia, reported as 75.3 ± 9.7 g cm^−3^. LaForge et al. [2] apparently took the listed Polyhymnia density at face value and characterized the asteroid as a compact ultradense object (CUDO) that “could be composed of high-Z superheavy and “condensed” α-nuclei matter elements beyond the known periodic table”.

There might be islands of stability for superheavy elements (SHEs) at *Z* = 108, 114, 162, 164, and/or 184 [3–6]. They may have closed nuclear shells or conform to deformed shell stabilization; some could have relatively long half-lives (perhaps ranging from milliseconds up to a few hours) compared to neighboring elements in the periodic Table. However, to date no SHEs have been identified in nature or synthesized in the lab [7, 8].

Kiren et al. [9] also apparently accepted the listed density of (33) Polyhymnia (~ 75 g cm^−3^) and proposed that the asteroid could be composed of degenerate dark matter—hypothetical materials composed of densely packed fermions affected by quantum degeneracy pressure. They calculated that a body composed of degenerate dark matter with the listed mass and radius of (33) Polyhymnia would have a density of ~ 73 g cm^−3^—close to the value listed by Carry [1]. However, the nature of dark matter is unknown; it might not even consist of fermions [10, 11], in which case it would not obey the Pauli exclusion principle or be subject to degeneracy pressure.

## Results and discussion

### The chemical composition of solar system bodies

**Fig. 1:**
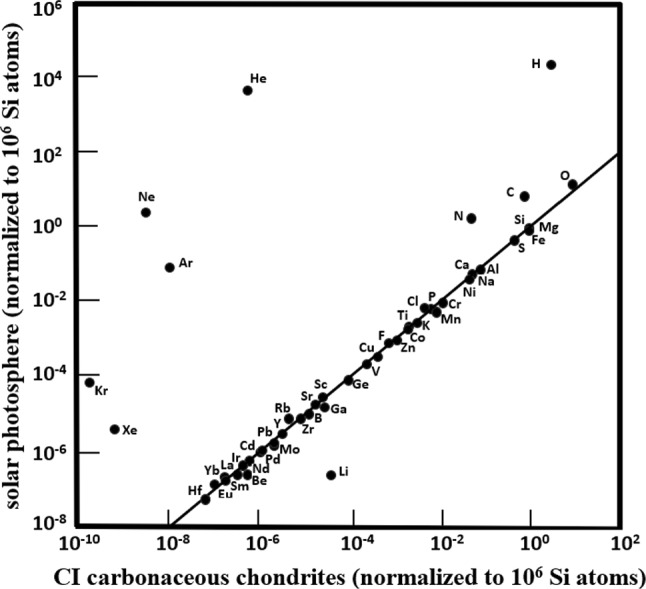
Log–log plot of selected elemental abundances in the solar photosphere versus those in CI carbonaceous chondrites. The photosphere/CI chondrite ratios for most elements are close to unity; these elements include refractory, common and volatile elements with lithophile, siderophile, and chalcophile affinities. Noble-gas abundances are much higher in the Sun than in CI chondrites because they did not quantitatively condense into solids. Diagram redrawn from Rubin and Ma [19] modified from McSween and Huss [17] and Dye et al. [20]

With the exceptions of noble gases (He, Ne, Ar, Kr, Xe) and highly volatile elements (C, H, N, O) that do not fully condense into solids, and Li, Be, and B (which are destroyed within the Sun), most naturally occurring elements have approximately the same relative abundances in the solar photosphere as they do in CI carbonaceous chondrite meteorites [12] (Fig. 1). The Earth has about the same chondritic interelement ratios of non-volatile elements as CI chondrites [13–15]; so do other planets [16]. Asteroids are categorized either as (1) unmelted bodies with chondritic bulk chemical compositions throughout their interiors or (2) differentiated or partly differentiated objects, formed by extensive-to-global melting of chondritic asteroid progenitors [17, 18].

The 25 most abundant elements in the Solar System are listed in Table 1, normalized to one atom of Si. These 25 elements constitute > 99.999% of the matter in the Solar System. If the elements that remained largely to completely in the gas during condensation are excluded (i.e., the noble gases and highly volatile elements (C, H, N, O)), then the 10 most abundant non-volatile elements are: Mg, Si, Fe, S, Al, Ca, Na, Ni, Cr, and Mn. At the oxygen fugacities pertaining to ordinary chondrites (the most common meteorites), Mg, Si, Al, Ca, Na, Cr, and Mn partition into silicate and oxide minerals, S partitions into sulfides, Ni partitions into metallic phases, and Fe partitions into silicates and oxides (as Fe^2+^ and Fe^3+^), and metal (as Fe^0^).

**Table 1:** 25 most abundant elements in the Solar System, normalized to one atom of Si

H	28,200	N	2.63	Ar	0.112	Cr	0.0135	Ti	0.0024
He	2820	Mg	1.023	Al	0.0851	Mn	0.00933	Co	0.00229
O	20.9	Si	1.0	Ca	0.0646	P	0.00525	Zn	0.00126
C	10.0	Fe	0.891	Na	0.0575	Cl	0.00525	F	0.000832
Ne	3.31	S	0.447	Ni	0.0501	K	0.00372	Cu	0.000524

Data from Table 2.2 of Lodders and Fegley [21]

### Meteorites and asteroids

Nearly 79,000 meteorites have so far been classified. Approximately 800 are from the Moon and ~ 420 from Mars. The remaining ~ 98.5% of meteorites are from asteroids. These asteroidal meteorites can be divided into the structural categories of stones (consisting of major silicates, oxides, sulfides, and metallic Fe–Ni–Co, with minor organic matter, phosphate and phosphide, carbonate, carbide and elemental C, and traces of other components), irons (mainly metallic Fe–Ni–Co, sulfide, phosphide, graphite, and accessory to trace amounts of carbides, nitrides, and other phases), and stony-irons (containing ~ 50 wt% silicates and associated phases and ~ 50 wt% metallic Fe–Ni–Co and sulfide).

There are numerous links between asteroids and meteorites:

1. The metallographic cooling rates of meteorites (1–100 K Ma^−1^) suggest derivation from bodies a few hundred kilometers in diameter [22, 23], in the size range of about two-dozen large asteroids.
2. The presence of solar-wind-implanted noble gases in meteorite regolith breccias [24] shows they were derived from bodies too small to retain significant atmospheres.
3. The old formation ages of most meteorites (~ 4.56 Ga) indicate their parent bodies were small and cooled very early in Solar System history [25].
4. The cosmic-ray exposure (CRE) ages of many stony meteorites are tens of millions of years [26], consistent with the time inferred for bodies in the asteroid belt to reach Earth.
5. The orbits of more than 1000 fireballs, including dozens that dropped recovered meteorites, are very similar to those of Earth-crossing asteroids [27, 28].
6. The reflectance spectra of asteroids show they are composed of common materials; many match particular varieties of meteorites after space weathering is taken into account [18].
7. The brecciated nature of many meteorites is consistent with the extensive impact-cratering observed on imaged asteroids [24, 29, 30].
8. Samples returned from asteroids closely resemble those of meteorites: (25143) Itokawa, LL chondrites [31]; (162173) Ryugu, CI chondrites [32]; (101955) Bennu, CI chondrites [33].
9. The *Dawn* spacecraft confirmed the composition and mineralogy of asteroid (4) Vesta match those of HED (howardite–eucrite–diogenite) achondritic meteorites [34].
10. Four asteroids observed telescopically before colliding with Earth yielded common meteorites: 2008 TC_3_, mainly ureilites and carbonaceous chondrite materials [35, 36]; 2018 LA, howardite [37]; 2023 CX_1_, L5-6 chondrite [38, 39]; 2024 BX_1_, aubrite [40]. [Other small asteroids observed before impact (2014 AA; 2019 MO; 2022 EB5; 2022 WJ1; 2024 RW1; 2024 UQ) have not yielded recovered meteorites.]

These 10 links confirm the proposition that asteroids are composed of the same materials as meteorites.

The approximate upper limit on the density of an asteroid would be that of a non-porous, unfractured metallic body composed of ~ 90 wt% Fe (*ρ* = 7.86 g cm^−3^) and ~ 10 wt% Ni (*ρ* = 8.91 g cm^−3^). [The typical Fe/Ni weight ratio of iron meteorites is 11.4 [41]]. An asteroid with a composition in this range would likely be a fragment of the metallic core of a differentiated body and would have a bulk density of ~ 8 g cm^−3^. Any reported asteroid densities higher than that are unrealistic.

Although some metallic elements have appreciably higher densities than Fe and Ni (e.g., W, 19.35 g cm^−3^; Re, 21.04 g cm^−3^; Os, 22.6 g cm^−3^; Ir, 22.4 g cm^−3^; Pt, 21.45 g cm^−3^), the concentrations of these refractory siderophile elements in meteorites are very low. For example, the iron meteorite with the highest measured Ir concentration is Avce, a member of the IIAB magmatic iron-meteorite group. This group was derived from a differentiated asteroid and was part of the metallic core that underwent fractional crystallization [41]. Avce contains a total of < 0.05 wt.% refractory siderophile elements: e.g., 4.00 µg/g W, 7.309 µg/g Re, 59.5 µg/g Ir, and 39.4 µg/g Pt [42] plus roughly comparable concentrations of Mo, Ru, Rh, Pd, and Os. No non-metallic components were identified in Avce [43].

Many asteroids of the S-type taxonomic class (such as (33) Polyhymnia) are likely composed of ordinary-chondrite (OC) material [44, 45]. These are the most common meteorites, constituting 78% of well-classified observed falls. There are three OC groups: H—high total iron; L—low total iron; and LL—low total iron, low metallic iron. Ordinary chondrites contain major silicates (olivine ((Mg,Fe)_2_SiO_4_), low-Ca pyroxene ((Mg,Fe)SiO_3_), diopside (CaMgSi_2_O_6_), plagioclase ((Na,Ca)(Si,Al)_3_O_8_)), metallic Fe–Ni, and troilite (FeS), minor chromite (FeCr_2_O_4_), and phosphates (Ca_5_(PO_4_)_3_Cl; Ca_9_NaMg(PO_4_)_7_)), and non-exotic accessory phases (e.g., ilmenite (FeTiO_3_), rutile (TiO_2_)). The mean bulk densities of ordinary chondrite falls decrease from H (3.35 ± 0.01 g cm^−3^) to L (3.30 ± 0.01 g cm^−3^) to LL (3.18 ± 0.02 g cm^−3^) [46]. The low standard deviations (1 sigma) show that no ordinary chondrites have densities remotely approaching 75 g cm^−3^.

### Uncertainties in asteroid density determinations

Asteroid densities are not measured directly. They are determined by dividing mass measurements (with varying uncertainties) by volume (which is itself calculated from diameter and shape estimates). As pointed out by Kiren et al. [9], Polyhymnia’s mass of (6.20 ± 0.74) × 10^18^ kg (with zero porosity) was determined by assessing its gravitational pull on other small bodies. But because the gravitational pull of small bodies is slight, there can be great uncertainties in mass and density estimates [1]. Such uncertainties can lead to unrealistically high densities such as 75.3 ± 9.7 g cm^−3^ for (33) Polyhymnia. In fact, Tang et al. [47] subsequently determined a lower mass for (33) Polyhymnia ((1.03 ± 0.40) × 10^18^ kg) after its close encounters with other asteroids. This lowers Polyhymnia’s calculated bulk density to 12.5 g cm^−3^, a less fantastic, but still unrealistically high value.

Carry [1] compiled mass and volume estimates of 287 small bodies including asteroids, comets, and trans-Neptunian objects (TNOs). He noted that the given uncertainties in mass determinations should be considered lower limits and that ~ 30% of the measurements have uncertainties of at least 50%. He ranked the calculated bulk densities on their degree of reliability, ranging downward from A to E: A and B—estimates with precision uncertainties < 20% (with A associated with a higher number of estimates or with a spacecraft encounter); C—estimates with uncertainties between 20 and 50%; D—uncertainties between 50 and 100%; E—uncertainties exceeding 100%. Sixty-five entries were marked with a cross (*X*) and designated “unrealistic,” “irrelevant,” and “non-physical.” One asteroid marked with a cross is (1686) De Sitter with a listed bulk density of 450.51 ± 220.97 g cm^−3^.

The mean bulk density of the 50 main belt asteroids and Near-Earth objects ranked with an A or B (the most reliable estimates) is 2.37 ± 1.13 g cm^−3^; the median value is 2.18 g cm^−3^. In contrast, the bulk density of asteroid (33) Polyhymnia is listed as 75.3 ± 9.7 g cm^−3^ and ranked with a cross. This value was specifically characterized as unrealistic and was not intended to be accepted as an accurate measurement of the asteroid’s density. Nonetheless, LaForge et al. [2] apparently took the listed Polyhymnia density at face value and classified the asteroid as a compact ultradense object (CUDO) that “could be composed of high-Z superheavy and “condensed” α-nuclei matter elements beyond the known periodic table.”

### Reflectance spectra of “CUDO” asteroids

**Fig. 2:**
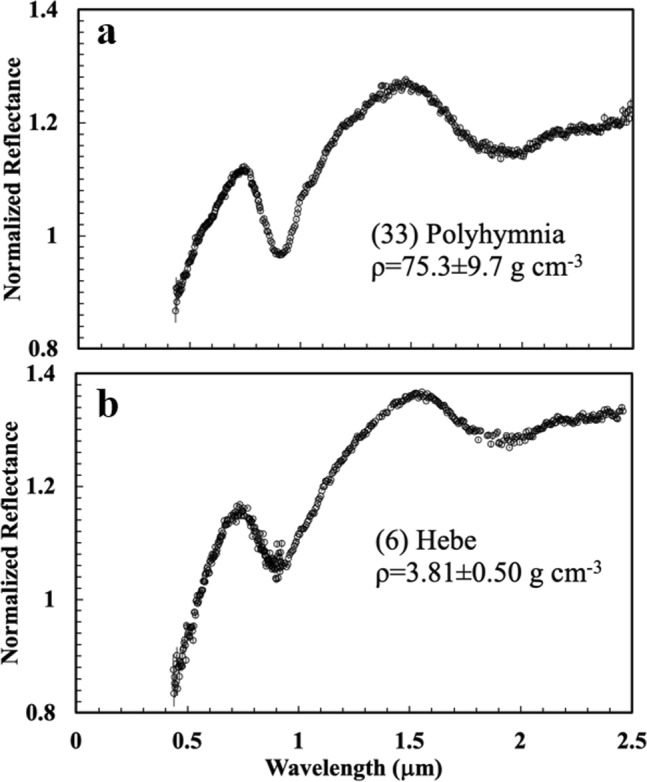
Reflectance spectra of asteroids (**a**) (33) Polyhymnia [47] and (**b**) (6) Hebe [51]. Both are S-type asteroids with very similar spectra and are assumed to be composed of very similar material. Nevertheless, the bulk densities listed by Carry [1] shown in the figure are wildly divergent. This is due entirely to the high uncertainties in the diameter and mass of Polyhymnia. The error bars are one sigma

The reflectance spectra of asteroids with unrealistically high bulk densities are not anomalous. These asteroids appear similar to others of their taxonomic class. For example, (33) Polyhymnia has a reflectance spectrum (Fig. 2a) in the visible and near-infrared wavelength regions with absorption bands characteristic of a mixture of pyroxene and olivine [48, 49]—common components of ordinary chondrites. The spectrum is nearly identical to that of another S-type asteroid: (6) Hebe (Fig. 2b), which does not have an anomalous bulk density (*ρ* = 3.81 ± 0.50 g cm^−3^ [1]). Hebe has been linked to the H-chondrite meteorites due to similarities in the Band Area Ratio (ratio of the areas of the two absorption bands at ~ 0.9 and ~ 1.9 μm, respectively) and the asteroid’s location near the 3:1 meteorite-supplying, mean-motion resonance with Jupiter [50].

### Apparent absence of exotic matter in the solar system

The unrealistically high density of asteroid (33) Polyhymnia, as well as several others listed in Carry [1] [e.g., (152) Atala, 47.92 ± 13.10 g cm^−3^; (675) Ludmilla, 73.99 ± 15.05 g cm^−3^] do not constitute evidence that any of these bodies are CUDOs (compact ultradense objects). There is no evidence from these irrelevant, non-physical density values that some asteroids contain strange matter (likely confined to neutron stars [52, 53], alpha matter (alpha particles in a Bose–Einstein condensate [54] which may occur naturally only in extreme astrophysical environments [55], stable SHEs (which, to date, have not been shown to exist), or degenerate dark matter [9] (which also has not yet been shown to exist).

## Conclusions

Asteroids categorized as compact ultradense objects (CUDOs) are mischaracterized because of the acceptance of anomalous density values specifically flagged as unrealistic, irrelevant, and non-physical. The reflectance spectra of these bodies show they are typical members of their taxonomic class. These asteroids are made of common materials—mainly silicates, metallic Fe–Ni, sulfides, oxides, and organic matter. The upper limit on the density of an asteroid is ~ 8 g cm^−3^ for a non-porous core fragment composed of ~ 90 wt% Fe and ~ 10 wt% Ni. Stable superheavy elements (SHEs) have not been shown to exist in nature or in the lab. Degenerate dark matter has also not been shown to exist. Alpha matter (alpha particles in a Bose–Einstein condensate) may occur only in extreme astrophysical environments. Exotic matter does not occur in asteroids.

## Data Availability

There are no data associated with this manuscript.
